# Vulvar cancer: surgical management and survival trends in a low resource setting

**DOI:** 10.1186/s43046-019-0015-y

**Published:** 2020-01-14

**Authors:** Navin Kumar, Mukur Dipi Ray, D. N. Sharma, Rambha Pandey, Kanak Lata, Ashutosh Mishra, Durgesh Wankhede, Jyoutishman Saikia

**Affiliations:** 1 Department of Surgical Oncology, Dr. BRA-IRCH, All India Institute of Medical Sciences, New Delhi, India; 2 Department of Radiation Oncology, Dr. BRA-IRCH, All India Institute of Medical Sciences, New Delhi, India; 3 Department of Nuclear Medicine, All India Institute of Medical Sciences, New Delhi, India

**Keywords:** Vulvar cancer, Modified radical vulvectomy, Inguinofemoral node dissection, Multi-disciplinary tumor board, Survival

## Abstract

**Background:**

This study aims to analyze risk factors, clinical profiles, treatment protocols, and disease outcomes in histologically proven resectable vulvar cancer (VC) patients according to tumor stage. This is a retrospective analysis of a prospectively collected database of 20 VC patients from May 2014 to June 2019.

**Results:**

The mean age of VC diagnosis was 55 years, with a range of 38–84 years. The incidence was four cases per year. The disease incidence was significantly more in post-menopausal (65%) and multiparous (90%) women. According to FIGO staging of vulvar cancer, stages I, II, and III were assigned to 6, 1, and 11 patients respectively. Two patients suffered from stage IVa vulvar melanoma. All patients had undergone surgical interventions. Patients treated with only nonsurgical (chemotherapy/radiotherapy/chemo-radiotherapy) treatment modalities were excluded from the study. Fifteen patients were treated with wide local excision (WLE), bilateral inguinofemoral dissection (B/L IFLND), and primary repair. Four and one patients were treated with radical vulvectomy (RV) and modified radical vulvectomy (MRV) [with or without B/L IFLND and PLND] respectively. Reconstruction with V-Y gracilis myocutaneous and local rotation advancement V-Y fasciocutaneous flaps were done in two patients. Therapeutic groin nodal dissection was performed in 19 patients except in one patient who was treated by palliative radical vulvectomy. In the final histopathology reports, tumor size varies from 0.5 to 6.5 cm (mean 3.35 cm) with the predominance of squamous cell carcinoma (18 out of 20 patients). Only 10 out of 18 eligible patients received adjuvant treatment. Poor patient compliance has been one of the major reasons for adjuvant treatment attrition rate. Systemic and loco-regional metastasis occurred in 3 patients each arm respectively. Poor follow up of patients is the key limitation of our study.

**Conclusion:**

Vulvar cancer incidence was significantly high in post-menopausal and multiparous women. The most important prognostic factors were tumor stage and lymph node status. Oncological resection should be equated with functional outcome. The multidisciplinary team approach should be sought for this rare gynecological malignancy.

## Background

As per GLOBOCAN (Global Cancer Incidence, Mortality and Prevalence) 2018 data, Vulvar cancer was placed at the 33rd rank among all new cases in India [1]. Carcinoma vulva is a rare disease and it consisted of 4% of all gynecological malignancies [2]. This is more prevalent in post-menopausal multiparous women. The increased age is itself a high-risk factor. The most common symptoms are pruritus, ulcer, vaginal discharge, or pain. Diagnosis is inferred by vigilant history, clinical examinations, vulvar biopsy, and/ or diagnostic imaging. The most common histology is squamous cell carcinoma followed by melanoma, basal cell carcinoma, and adenocarcinoma [2]. The staging of vulvar cancer is popularly done according to the International Federation of Gynecology and Obstetrics (FIGO) [3]. The treatment depends on the disease histology, stage, and patient’s performance status, which consists of surgery, chemotherapy, radiotherapy, and palliative supportive care. Lymph node positivity is an independent bad prognostic factor [3]. According to the disease’s stage, 5 years survival rates range from 86% for early-stage disease (FIGO stage I) to 19% for metastatic disease (FIGO stage IVB) and lifetime risk of developing vulvar cancer is 0.3% [4]. Being a rare gynecological tumor, there is a paucity of literature data for changing trends in management protocols, disease outcomes, and long-term survival data. We present our institute’s retrospective collected data from the prospectively maintained database with informed written consent concerning risk factors, treatment protocols, disease outcome, and survival data of VC patients over 5 years.

## Methods

The case records from a prospectively maintained database of 20 vulvar cancer patients treated between May 2014 and June 2019 were reviewed. We analyzed the demographic profile, the disease incidence, clinical details, diagnosis, stage, treatment modalities used, disease outcome, and survival data**.** Patients were evaluated in a dedicated gynecological cancer disease management group (DMG). It consisted of surgical oncologists, medical oncologists, radiologists, pathologists, onco-anesthesiologist, radiation oncologist, physiotherapist, dietitian, and palliative care clinicians. Each patient was subjected to various basic hematological, radiological, and pathological investigations. Computed tomography scan (CT scan)/magnetic resonance imaging (MRI)/positron emission tomography-computed tomography (PET-CT) along with cysto-sigmoidoscopy were advised in selected patients. The staging was assigned according to the new updated American Joint Committee on Cancer (AJCC) Tumor-Node-Metastases (TNM) staging and the International Federation of Gynaecology and Obstetrics (FIGO) surgical staging systems for carcinoma of the vulva [3]. Treatments were advocated according to the disease's stage, histology, performance status, and the possibility of achieving tumor-free resection (R0 resection). Final histopathology reports were reviewed in DMG followed by stage-based treatment protocol. Post-treatment regular follow-up was advised as per our institutional protocol.

### Statistical analysis

**Fig. 1:**
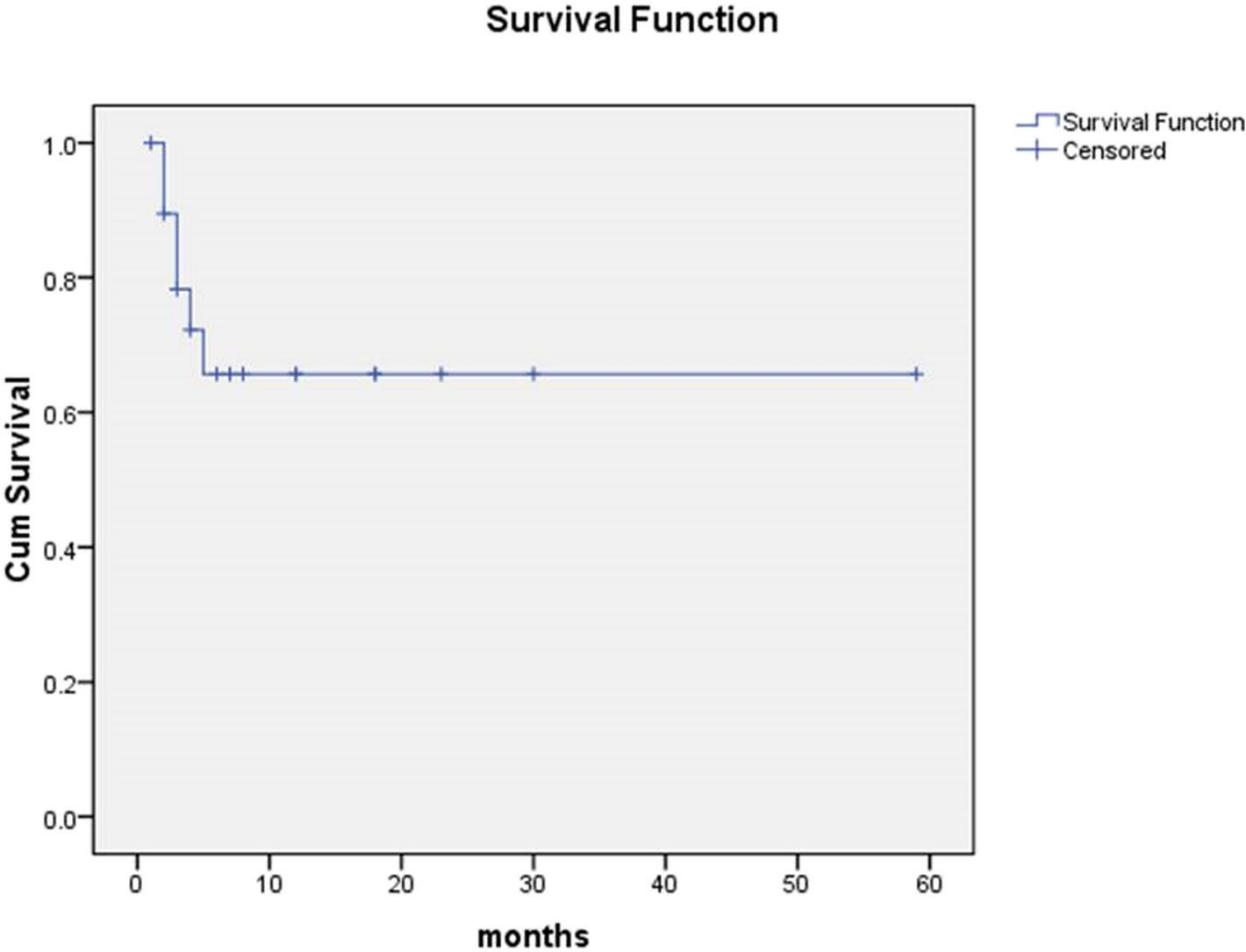
Kaplan-Meier survival curve showing 5 years disease-free survival

The obtained data from prospectively maintained computerized databases were coded, tabulated, and analyzed using SPSS package version 12 (IBM Corporation) and were analyzed using descriptive and inferential statistics based on objectives of the study with written informed consent. Statistical analysis for survival was demonstrated in the Kaplan-Meier curve (Fig. 1).

### Follow-up

Follow-up time varied from 1 month to 59 months, with a mean follow-up time of 11.1 months.

## Results

The range of VC patient’s age was 38–84 years with a median age of 55 years. The peak incidence was 50–70 years. The disease incidence was more common in post-menopausal (*n* = 13) as compared to premenopausal (*n* = 7) women and in multiparous (*n* = 18) than nulliparous women (*n* = 2). Average 4 VC patients were seen per year in the outpatient clinic. Most of the patients were from low to modest socioeconomic status. The demographic profile with clinical details is shown in Table 1. The most common sub-type of vulvar cancer was squamous cell carcinoma (90%), including one adenosquamous carcinoma. Two patients (10%) were diagnosed with malignant melanoma. The most common subsite of disease occurrence was labia majora (85%). Remaining 3 patients (15%) had disease epicenter in labia minora. The most common FIGO stage at presentation in our case series was stage III (*n* = 11) followed by FIGO II (*n* = 6) and I (*n* = 1). The remaining two patients were diagnosed as stage IVA malignant melanoma of vulva. Seventeen patients had unilateral labial involvement while three had bilateral involvement. Five patients had unilateral inguinal lymphadenopathy while six had bilateral inguinal lymphadenopathy. None of the patients had ulcerated or fixed matted inguinal nodes or clinically palpable pelvic nodes.

**Table 1:** Demographic and clinical profiles of vulvar cancer patients

	Number of cases	Percentage of cases (%)
Age range		
30–39	1	5.0
40–49	6	30.0
50–59	5	25.0
60–69	5	25.0
70–79	2	10.0
≥ 80	1	5.0
Religion		
Hindu	18	90.0
Muslim	1	5.0
Christian	1	5.0
Menopause status		
Pre-menopausal	13	65.0
Post-menopausal	7	35.0
HIV status		
Positive	1	5.0
Negative	19	95.0
Parity		
Multiparous	18	90.0
Nulliparous	2	10.0
Medical comorbidity		
Diabetes mellitus	3	15.0
Hypertension	5	25.0
No medical comorbidity	12	60.0

Correlation of clinical features, disease characteristics, treatment protocols, disease status, and survival outcome are illustrated in Table 2. Wide local excision (WLE) with bilateral inguinofemoral node dissection (B/L IFLND) and primary closure were the most common surgical procedures performed (15 out of 20 patients). The other five patients were treated with radical vulvectomy (RV) (*n* = 4) and modified radical vulvectomy (MRV) (*n* = 1). Among these, one patient was treated before with neoadjuvant concurrent chemo-radiotherapy (CCRT), 25 fractions of 50 Gy over 5 weeks with cisplatin followed by RV. Groin nodal dissections (IFLND) were performed in all patients except the one who had undergone palliative resection with wide local excision, because of poor performance status of the patient having ulcerated and fungated vulvar growth. The decision of IFLND was done as per institutional protocol based on preoperative imaging findings, disease presentation at an advanced stage, nonadherence of patients to regular follow-up and minimal additional morbidity with groin nodal dissection by Ray’s River flow incision technique. Node positivity in the final histopathology was found in 13 out of 19 patients. Ray's River flow incision technique was used for ilioinguinal nodal dissection to minimize the surgical morbidities, especially the flap necrosis [5, 6]. Pelvic lymph node dissection (PLND) was done along with IFLND in three selected patients, having grossly enlarged suspicious deep inguinal nodes with criteria like size >/= 1 cm, round-shaped and hard in consistency. Reconstruction surgery was planned after careful intra-operative assessment of the defect after primary surgery and it was successfully executed in two patients with V-Y gracilis myocutaneous flap and local rotation advancement V-Y fasciocutaneous flap. Primary closure was achieved in the other eighteen patients. Modified radical hysterectomy with total vaginectomy and RV was done in one vulvar melanoma patient, given tumor infiltration to vagina with cervix. The post-surgical defect of this patient was reconstructed with V-Y gracilis myocutaneous flap. Partial wound dehiscence in the early post-operative period was managed with secondary suturing. In final histopathological specimen reports, tumor size varied from 0.5 to 6.5 cm, with a mean of 3.35 cm. The most common histology was squamous cell carcinoma (*n* = 18) followed by melanoma (*n* = 2). Histopathologically, well, moderately and poorly differentiated subtypes of squamous cell carcinoma were found in 4, 13, and 1 patients respectively. Two patients of vulvar malignant melanoma were diagnosed with amelanotic and nodular subtypes respectively. The outcomes of different treatment modalities were discussed thoroughly with the patients and the available treatment plans were mentioned. All patients underwent gross clinical R-0 resection, with microscopic positive margins in two patients. One had a deep positive margin, while another had multiple margins positivity. In five patients, margins were close (< 5 mm). Tumor depth was reported only in five cases, varying from 0.4 to 2.8 cm. Based on specimen histopathology, 18 out of 20 patients had actual indications for adjuvant treatment in view of advanced disease stage, nodal positivity, and/or close/positive margins. Eleven patients had stage III vulvar cancer while two had stage IV. Twelve patients had positive regional nodes. Because of noncompliance with patient and non-willingness for further adjuvant treatment, the adjuvant chemoradiotherapy (50.4 Gy, 28# with cisplatin) and radiotherapy (50.4 Gy, 28#) alone were advocated only in 8 and 3 patients. The surgical morbidities occurred in 7 patients. Three patients developed perineal/vulvar wound dehiscence in the post-operative period. These patients were treated with secondary suturing. Inguinal seroma and cellulitis occurred in 3 and 1 patients respectively. Seroma was dealt with multiple episodes of aspirations while cellulitis was managed with antibiotics and analgesics. There were two relatively unusual complications in the post-operative period. One patient developed a right inguinofemoral incisional hernia, which was treated with hernioplasty. Recto-vaginal fistula developed to another patient in the early postoperative period on the 21st day of surgery, which was treated curatively with staged surgical intervention. Intraoperatively there were dense adhesions in the pelvis with pus debris and a small rent in between the anterior rectal wall and posterior vaginal wall, which were managed with peritoneal lavage, excision of fistulous tract with primary closure, and a temporary diverting transverse colostomy. Stoma reversal was done after 8 weeks of prior surgery. Three patients defaulted in the post-operative period. The other three patients were not interested in further treatment apart from surgery due to poor family and social support. Six patients developed systemic (*n* = 3) and locoregional (*n* = 3) recurrences during the study follow-up period. Two patients developed bilateral lung metastasis, while the other had PET CT detected mediastinal and left supraclavicular nodal metastasis. Loco-regional recurrences occurred in three patients till the last follow-up and were varying from sites- perineum (*n* = 1), left inguinal region (*n* = 1) and vulvar surgical site (*n* = 1). One patient had a vulvar recurrence after 3 months of post-operative radiotherapy for close margin was treated with 2 cycles of cisplatin-based palliative chemotherapy. The patient lost to follow-up after the second cycle of chemotherapy. The mean follow-up was 11.1 months. Kaplan-Meier curve depicts that approximately 66% of patients had 5 years of disease-free survival (Fig. 1).

**Table 2:** Correlation of clinical features, disease characteristics, treatment, and disease status

Sl no.	Age (years)	Tumor location	Tumor size	Histology	Chemo/radiotherapy/def	Surgery	Stage	Lymph node	Disease status	F/U time (m)
1	42	U/L (R)	0.5 × 0.5	MM(AM)	ACRT	MRV+B/L IFLND	pT3N1M0(IVA)	3/28	RD	3
2	60	U/L (R)	0.5 × 0.5	SCC(WD)	ACRT	WLE+B/L IFLND	FIGO IIIB	7/22	RD	4
34	43	B/L	6.5 × 5.0	SCC(MD)	ACRT	WLE+B/L IFLND	FIGO IIIB	6/24	RD	3
	38	U/L (L)	5.0 × 3.5	SCC(MD)	ART	WLE+B/L IFLND+Recon*	FIGO IB	0/13	NED	23
5	40	B/L	0.5 × 0.5	SCC(MD)	NACRT	WLE+B/L IFLND	FIGO IIIB	1/7	NED	12
6	75	U/L (R)	2.5 × 2.5	SCC(PD)	ACRT	WLE+B/L IFLND	FIGO II	0/8	NED	18
7	75	U/L (L)	6.5 × 4.0	SCC(WD)	Def	WLE+B/L IFLND	FIGO IIIB	9/16	NED	3
8	60	U/L (L)	1.5 × 1.0	SCC(MD)	Def	RV+B/L (IFLND + PLND)+Recon^#^	FIGO IIIA	2/20	NED	2
9	57	U/L (R)	5.0 × 3.0	SCC(WD)		WLE+B/L IFLND	FIGO 1B	0/21	NED	59
10	66	U/L (L)	4.5 × 4.5	SCC(WD)	NWA	Palliative RV	FIGO IIIB	1/20	RD	2
11	44	B/L	6.5 × 6.0	SCC(MD)	Def	WLE+B/L IFLND	FIGO IIIA	0/13	NED	4
12	84	U/L (L)	5.0 × 4.0	SCC(MD)	ACRT	WLE+B/L IFLND	FIGO IB	2/18	NED	1
13	56	U/L (R)	3.5 × 2.0	SCC(MD)	NWA	WLE+B/L IFLND	FIGO IIIB	2/31	NED	30
14	64	U/L (R)	3.5 × 3.0	AS (MD)	NWA	RV + B/L (IFLND+PLND)	FIGO IIIB	1/17	NED	18
15	50	U/L (L)	1.5 × 1.0	MM(NM)	AC	RV+ MRH +B/L (IFLND+PLND)	pT3N1M0 (IVA)	0/12	RD	2
16	52	U/L (R)	4.0 × 2.0	SCC (MD) +VIN III		WLE+B/L IFLND	FIGO IA	9/17	NED	12
17	58	U/L (R)	2.0 × 1.5	SCC(MD)	ART	WLE+B/L IFLND	FIGO IIIC	0/9	NED	8
18	41	U/L (L)	3.0 × 1.5	SCC(WD)	ART	WLE+B/L IFLND	FIGO IB	0/12	NED	7
19	65	U/L (R)	2.0 × 1.0	SCC(MD)	ACRT	WLE+ I/L IFLND	FIGO IB	0/13	NED	6
20	44	U/L (R)	3.0 × 2.0	SCC(MD)	ACRT	WLE+B/L IFLND	FIGO IIIB	4/14	R	5

*Abbreviations***:** *U/L* unilateral, *B/L* bilateral, *L* left, *R* right, *SCC* squamous cell carcinoma, *MM* malignant melanoma, *NM* nodular melanoma, *AM* amelanotic melanoma, *AS* adenosquamous cell carcinoma, *WD* well-differentiated, *MD* moderately differentiated, *PD* poorly differentiated, *VIN* vulvar intraepithelial neoplasia, *ART* adjuvant radiotherapy, *ACRT* adjuvant chemoradiotherapy, *Def* defaulted case, *NWA* not willing for adjuvant treatment, *Pall Chemo* palliative chemotherapy, *WLE* wide local excision, *RV* radical vulvectomy, *MRV* modified radical vulvectomy, *MRH* modified radical hysterectomy, *IFLND* inguinofemoral lymph node dissection, *PLND* pelvic lymph node dissection, *NED* no evidence of disease, *RD* recurrent disease, *m* month, *FIGO* International Federation of Gynaecology and Obstetrics, *Recon* reconstruction

*Reconstruction by V-Y gracilis advancement flap

^#^Reconstruction by local rotation advancement V-Y fasciocutaneous flap

## Discussion

Prevalence and incidence of vulvar cancer in developing nations tend to have a relatively high as compared to that of the developed nations [1]. Squamous cell carcinoma is the most common histology of vulvar cancer. Other less common histological subtypes are extramammary Paget’s disease, melanoma, Bartholin’s gland tumors, adenocarcinoma, and basal cell carcinoma [7]. About two-thirds (65%) of our patients presented in advanced stage (FIGO stage III–IV). This figure corresponds to the same range as in the previously published literature [7].

Surgical management should be individualized. Even though the majority of the patient had undergone wide local excision with primary repair in our study, the margins had never been compromised and the oncological outcome had always been taken as a priority. The psychosexual sequelae and surgical morbidities associated with vulvar surgery and groin nodal dissection have driven treatment approaches to the more conservative ones. Only 10 patients were followed up beyond 6 months. The reason for poor follow-up could be due to poor patient compliance, as most of the patients were from low or modest socioeconomic status, uneducated and negligence. Clinical and histological nodal positivity were seen in 8 and 13 patients respectively. Among these, six patients developed either loco-regional (*n* = 3) or distant metastasis (*n* = 3) in a follow-up period. The present study suggests that the stage at presentation and lymph node positivity have poor prognostic values. Ipsilateral lymph node dissection is indicated for unilateral lesions, not crossing midline, and either negative ipsilateral nodes, or with positive lymphadenopathy with vulvar lesion smaller than 2 cm [8–10]. Also, the depth of invasion (DOI) was reported in only 2 patients histopathological reports. They had more than 6 mm DOI, along with few positive groin lymph nodes. The strict adherence to FIGO staging is important for disease prognostication and treatment outcome [11, 12].

In our study, nodal positivity was solely the most important bad predictive and prognostic factor; nevertheless, the final tumor stage, histology, the degree of differentiation, depth of invasion and lymphovascular invasion (LVI) also decide the survival outcome in literature [13–15]. The reason for the minimal inguinal and pelvic lymphadenectomy wounds morbidity could be due to Ray’s ‘River flow’ incision (two parallel curvilinear incisions) [5, 6] technique for ilioinguinal dissection. This may be contrary to the author Siller et al. [16], who had reported a major wound breakdown rate of 15–30%. In our study, adjuvant radiation was given based on lymph node metastasis, close surgical margin, size, and depth of the primary tumor. Out of 17 eligible patients for adjuvant treatment, only 11 patients had received it. The potential reasons for not getting treatment to the remaining six patients were defaulted follow-up and non-willingness due to logistic issues. Neoadjuvant radiotherapy/chemotherapy was not frequently practiced in our institutional setting that is why only one patient was treated with neoadjuvant chemoradiotherapy followed by surgery. However, the recent trend is shifting toward conservative surgery with the combined use of preoperative radiotherapy or chemo-radiotherapy [17–20]. The 5 years disease-free survival is 66%, which is comparable to the studies published by Sharma DN. et al. [13], Singh N. et al. [21], Rajshree D K. et al. [22], and Meelapkij P. et al. [23]. There are no large randomized controlled trials or meta-analysis because of the rarity of the disease. So, treatment guidelines are based on small retrospective individual center-based studies in the literature.

The majority of the patients present in the advanced stage in developing countries due to social stigma, low to middle socioeconomic status, low literacy rate, logistic issues, poor screening program, and insufficient awareness about the disease. Public awareness of warning symptoms of vulvar malignancy may help in early detection and cure. There is no current evidence for a specific screening of vulvar cancer. However, self-examination in women with lichen sclerosis advised for early detection of vulvar neoplasm [24]. Also, any patients with suspicious signs (e.g., pigmented lesions, irregular ulcers) or symptoms (e.g., chronic vulvar pruritus) should be early evaluated with skin biopsy [25]. Further research is warranted with large multicentric prospective randomized controlled trials to establish the definite screening guidelines, treatment protocols and survival outcome data for this rare gynecological malignancy in low-middle income countries.

## Conclusions

Vulvar cancer is a rare gynecological cancer, with a median age of 55 years and a peak incidence in fifth-seventh decades. Disease incidence was higher in multiparous and post-menopausal women. The multimodality treatment approach should be followed. Disease stage and lymph nodal positivity were the two most significant prognostic factors for survival in vulvar cancer. Adequate surgical resection with microscopic tumor-free margin should be the key concern. Oncological and functional outcomes should be balanced with meticulous surgical intervention.

## Data Availability

Data has been collected from the prospectively collected computerized database of the institution after getting the ethical clearance with a proper channel.

## Abbreviations

CT scan: Computed tomography scan
DMG: Disease management group
GLOBOCAN: Global Cancer Incidence, Mortality and Prevalence
LMIC: Low middle-income countries
LVI: Lymphovascular invasion
MDT: Multi-disciplinary tumor board
MRI: Magnetic resonance imaging
PET-CT: Positron emission tomography-computed tomography
SEER: Surveillance, Epidemiology, and End Results
TNM: Tumor-node-metastases
VC: vulvar cancer
